# Supplementary material for: Functional and structural diversity in deubiquitinases of the Chlamydia-like bacterium Simkania negevensis
Source: Nat Commun. 2023 Nov 13;14:7335. doi: 10.1038/s41467-023-43144-y (PMC10643670; doi:10.1038/s41467-023-43144-y)
Supplement: Supplementary file 1 — Supplementary Information [file 41467_2023_43144_MOESM1_ESM.pdf]

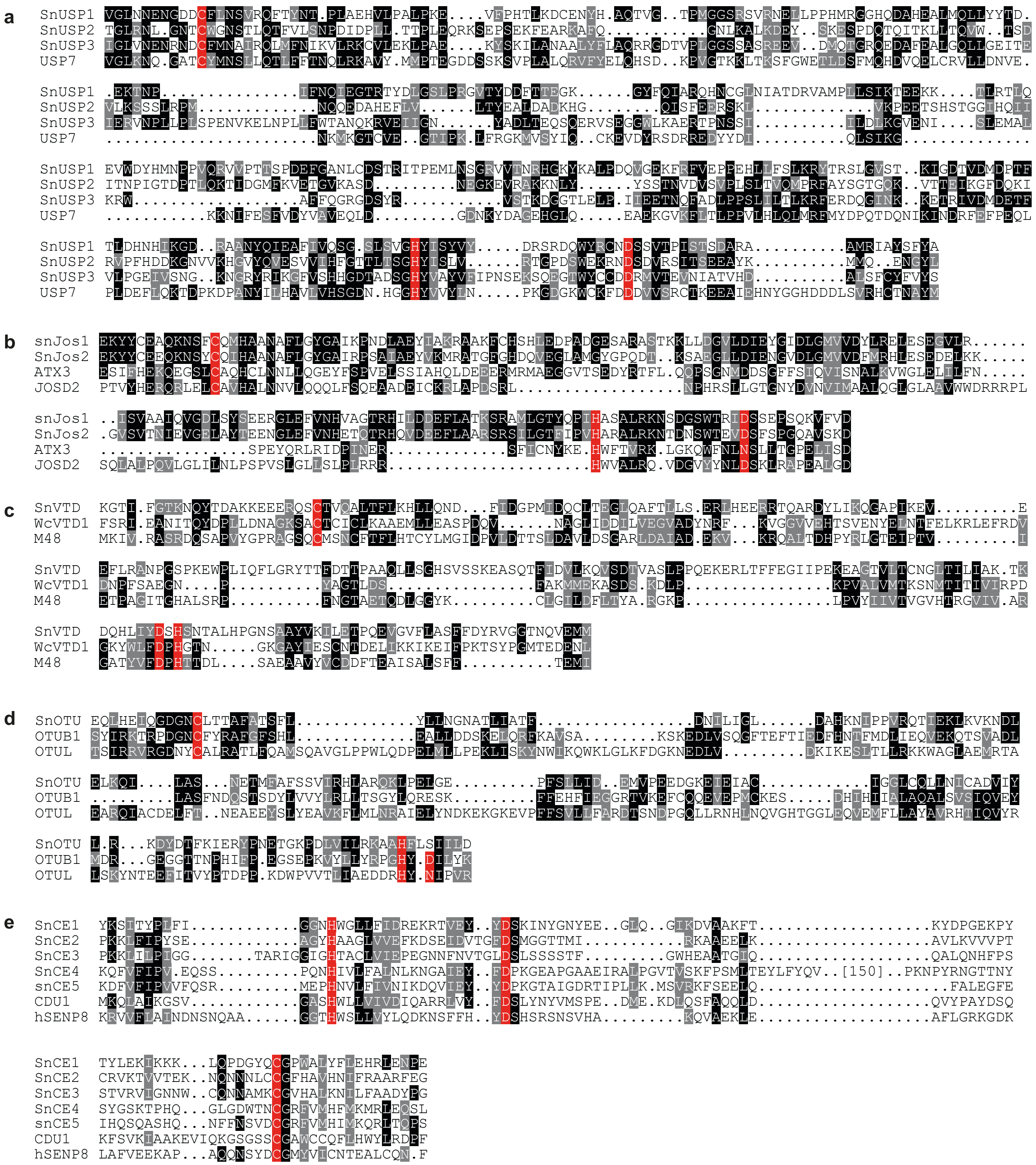

**Supplementary Figure 1**

Multiple alignment of deubiquitinase families with members from S. negevensis. The catalytic core domains of Simkania DUB candidates are aligned with representative members of the family using MAFFT. Positions that are invariant or conservatively replaced in at least 50% of the shown sequences are rendered on black or gray background, respectively. Catalytic residues are shown on red background.

a) Alignment of Simkania USP proteins SnUSP1, SnUSP2 and SnUSP3 with human USP7.

b) Alignment of Simkania Josephin proteins SnJos1 and SnJos2 with human ATX3 and JOSD2.

c) Alignment of Simkania VTD protein SnVTD with W. chondrophila WcVTD and herpesviral M48.

d) Alignment of Simkania OTU protein SnOTU with human OTUB1 and Otulin.

e) Alignment of Simkania CE proteins SnCE1, SnCE2, SnCE3, SnCE4 and SnCE5 with C. trachomatis CDU1 and human SENP8.

a

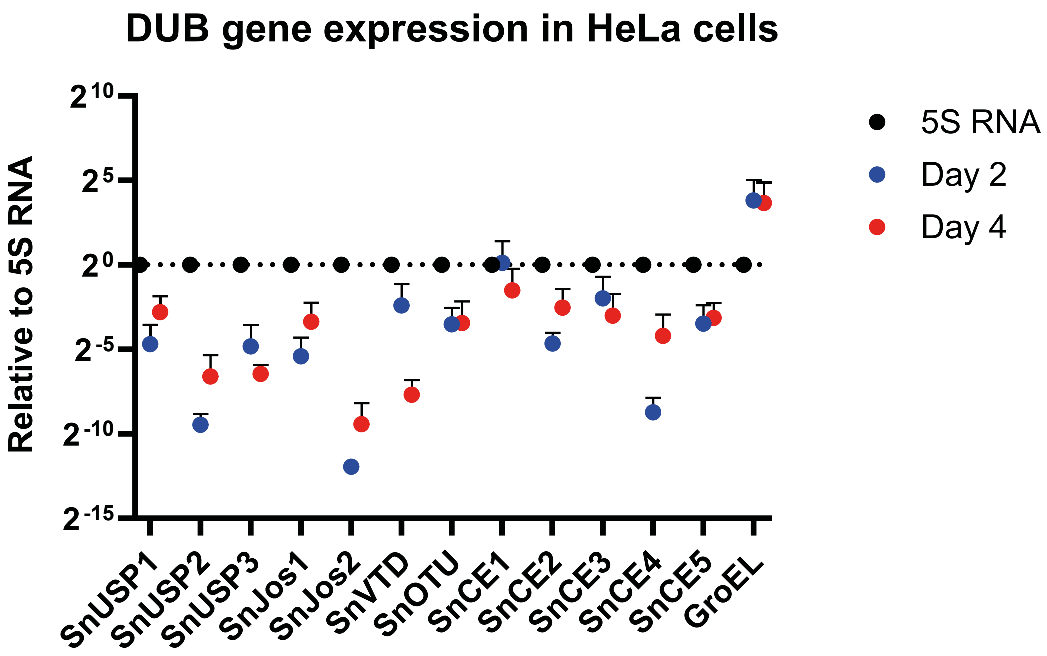


b

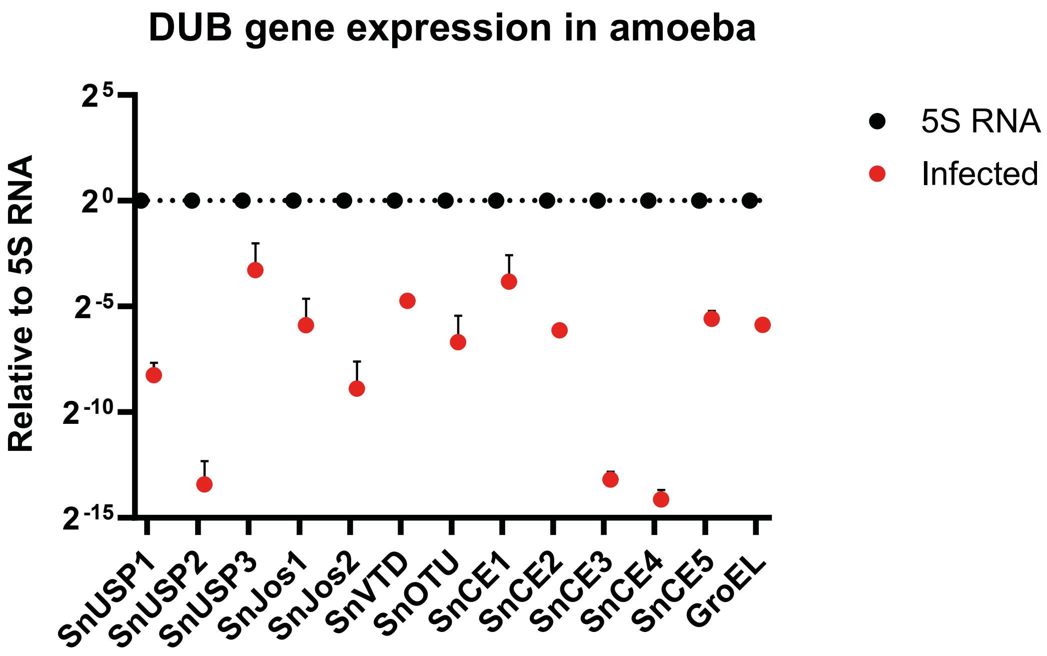


**Supplementary Figure 2**

a, Quantitative PCR analysis was performed on HeLa cells infected with *Simkania negevensis* on the second and fourth day after infection. The dots represent the mRNA quantities of the analyzed genes normalized against the 5S RNA of *Simkania negevensis*.

b, Quantitative PCR analysis was performed on *Acanthamoeba castellanii* continuously infected with *Simkania negevensis*. The dots represent the mRNA quantities of the analyzed genes normalized against the 5S RNA of *Simkania negevensis*.

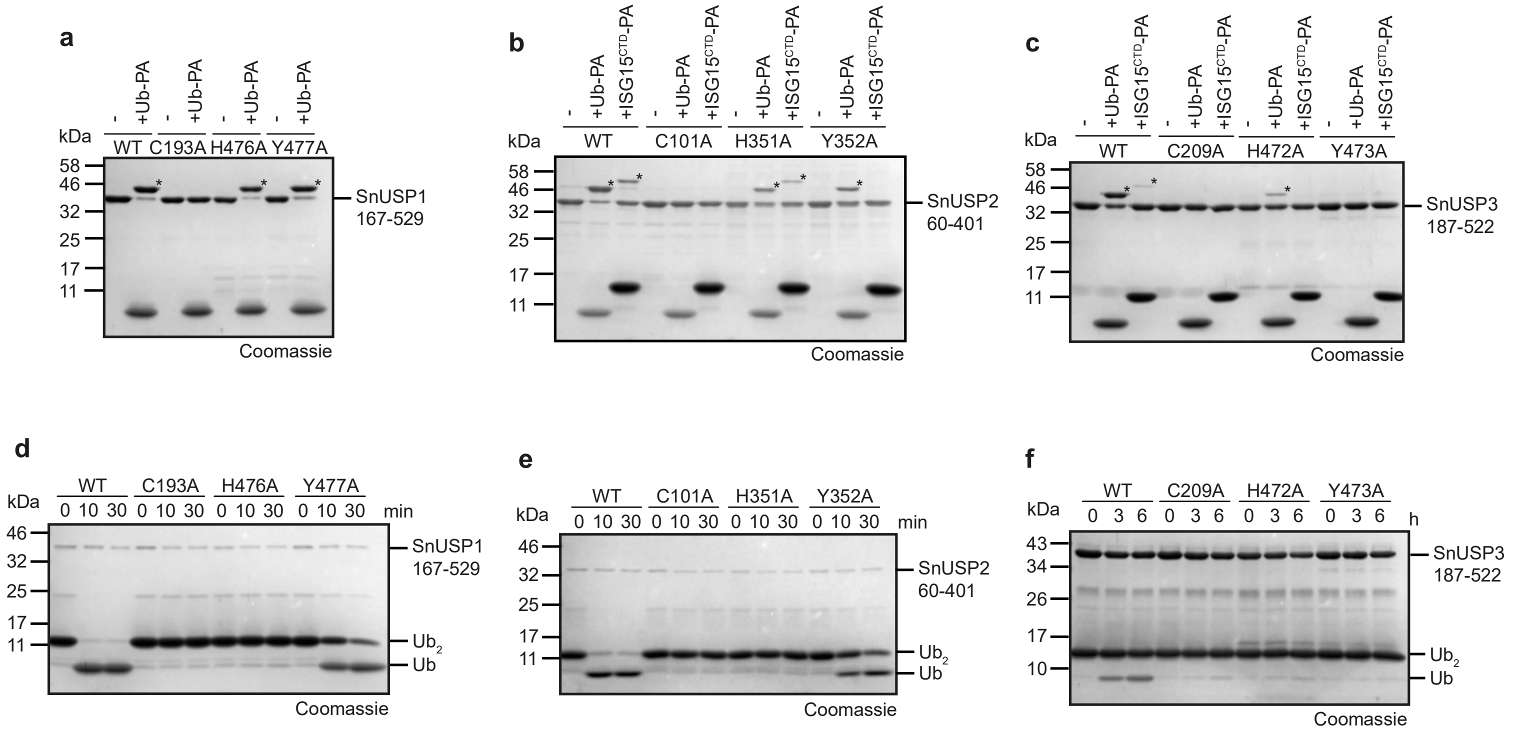


**Supplementary Figure 3**

a-c, Activity-based probe reaction of SnUSP1 (a), SnUSP2 (b) or SnUSP3 (c) comparing WT enzyme with catalytic and gatekeeper mutants. Asterisks (*) mark the shifted band after reaction.

d-e, Ubiquitin chain cleavage analysis. K48-linked di-ubiquitin chains were incubated with 0.5 μM WT enzyme or catalytic/gatekeeper mutants of SnUSP1 (d) or SnUSP2 (e).

f, Ubiquitin chain cleavage analysis. K63-linked di-ubiquitin chains were incubated with 5 μM WT enzyme or catalytic/gatekeeper mutant of SnUSP3.

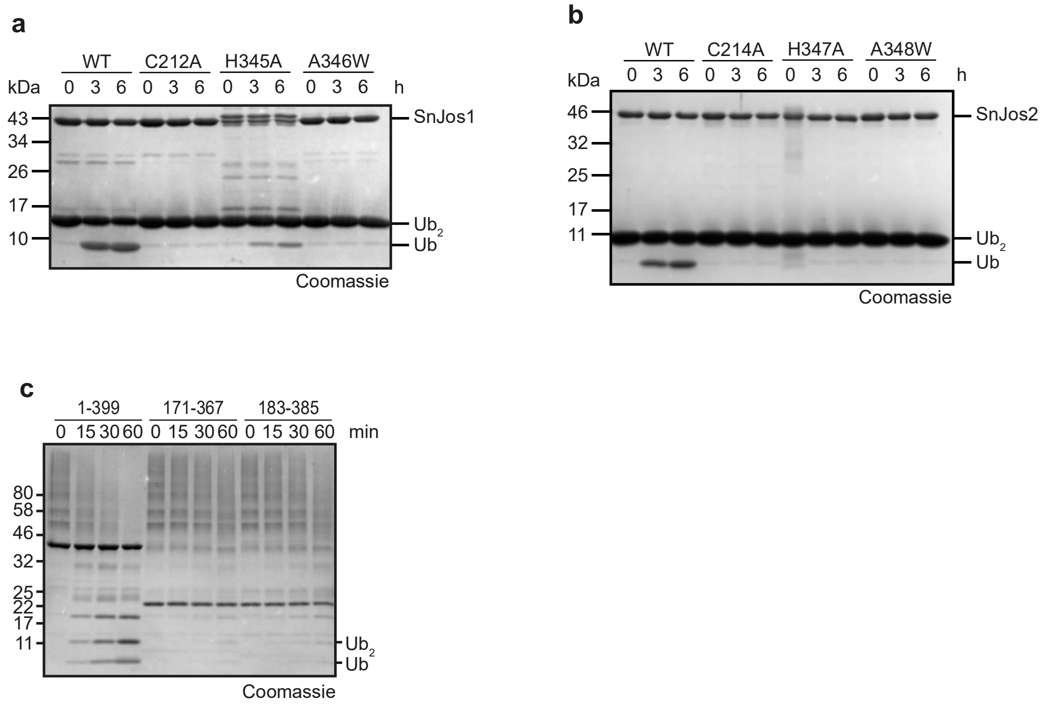


**Supplementary Figure 4**

a-b, Ubiquitin chain cleavage analysis. K11-linked di-ubiquitin chains were incubated with 5 µM WT enzyme or catalytic/gatekeeper mutants of SnJos1 (a) or SnJos2 (b).

c, Ubiquitin chain cleavage assay of SnJos1. Long K63-linked ubiquitin chains were incubated with the FL enzyme and compared to two truncated versions.

**a**

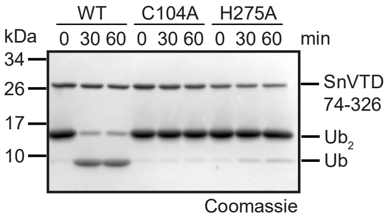


**Supplementary Figure 5**

a, Ubiquitin chain cleavage analysis. K6-linked di-ubiquitin chains were incubated with 5 μM SnVTD WT enzyme or catalytic inactive mutants.

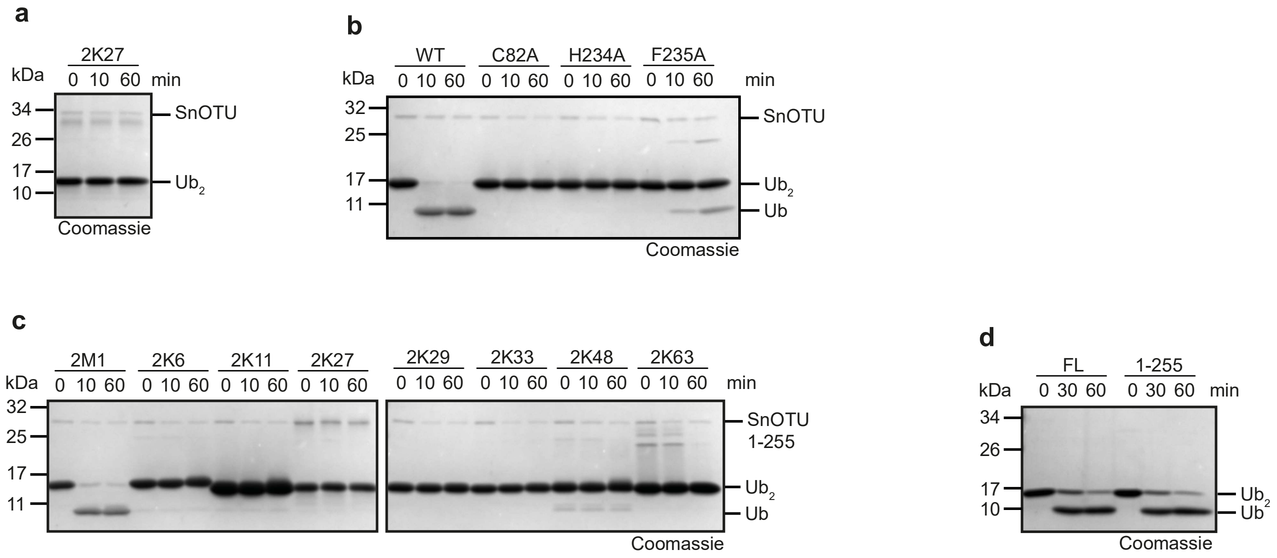


**Supplementary Figure 6**

**a-d,** Ubiquitin chain cleavage assays with SnOTU.

**a,** 0.5 µM SnOTU FL WT were incubated with K27-linked di-ubiquitin.

**b,** 0.5 µM SnOTU FL WT or catalytic/gatekeeper mutants were incubated with M1-linked di-ubiquitin.

**c,** 0.5 µM SnOTU truncation 1-255 were incubated with a panel of di-ubiquitins.

**d,** 0.1 µM SnOTU FL/1-255 were incubated with M1-linked di-ubiquitin.

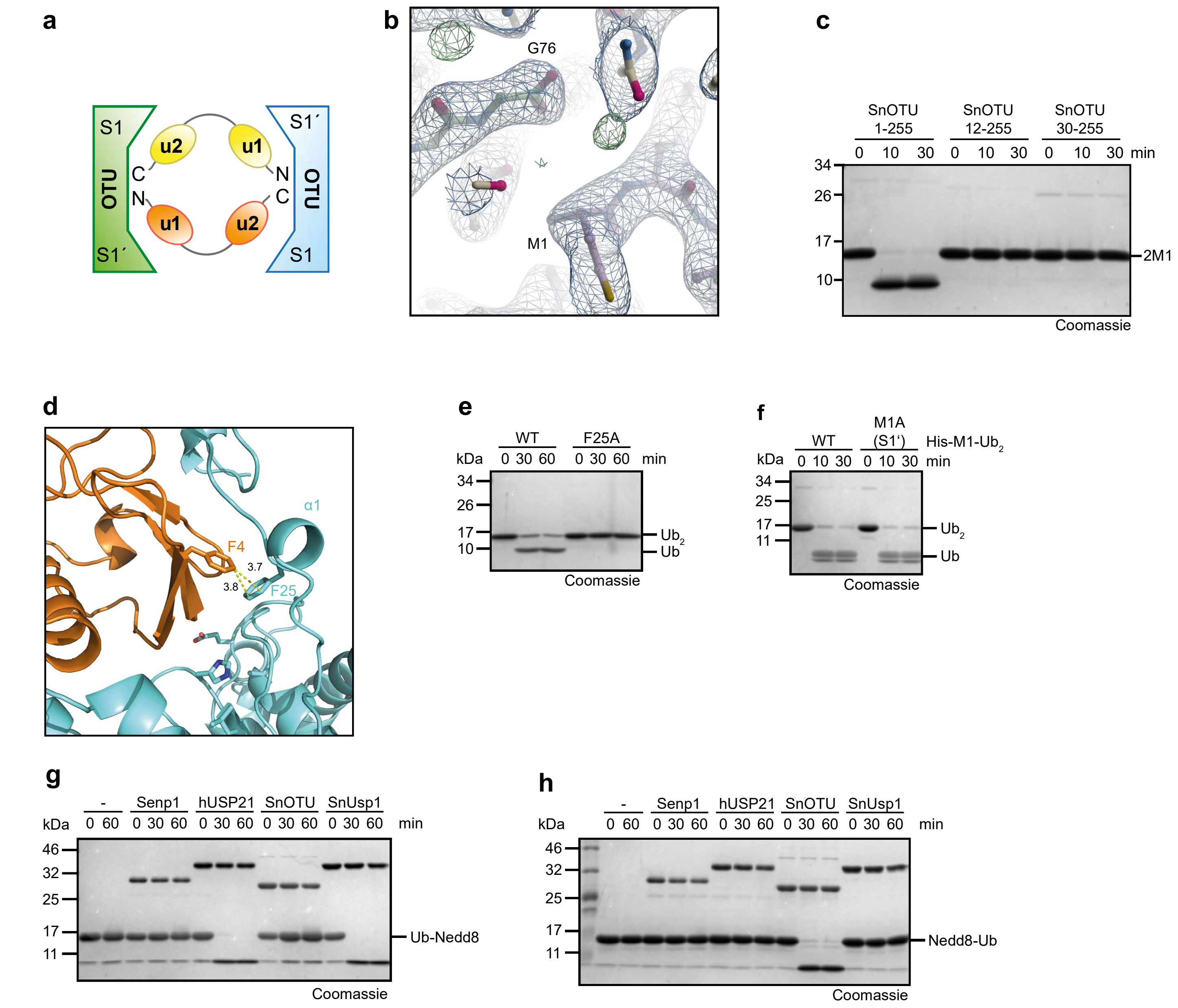


**Supplementary Figure 7**

a, Schematic representation of the solved complex structure consisiting of two SnOTU moieties with two crosslinking M1-linked di-ubiquitins.

b, 2Fo-Fc map contoured at 1 standart deviation above the mean showing the N- and C-termini of both ubiquitin chains in close proximity within SnOTUs active site.

c, Ubiquitin chain cleavage assay of SnOTU 1-255/12-255/30-255 with M1-linked di-ubiquitin. Enzymes were incubated with M1-linked di-ubiquitin.

d, Interactions between the Phe-4 of the S1‘ ubiquitin (colored orange) and
Phe-25 of SnOTU (colored teal). Residues involved in these interactions and acive site residues are shown as sticks. Interactions are indicated by yellow dotted lines.

e, Ubiquitin chain cleavage of 0.1 μM SnOTU WT and F25A mutant with M1-linked di-ubiquitin.

f, Activity of wildtype SnOTU against ubiquitin mutant. N-terminal His-tagged and mutated linear linked di-ubiquitin was incubated with 0.25 μM SnOTU for the indicated timepoints.

g, Ubiquitin-Nedd8 chimera (25 μM) cleavage assay with 5 μM of different enzymes.

h, Nedd8-Ubiquitin chimera (25 μM) cleavage assay with 5 μM of different enzymes.

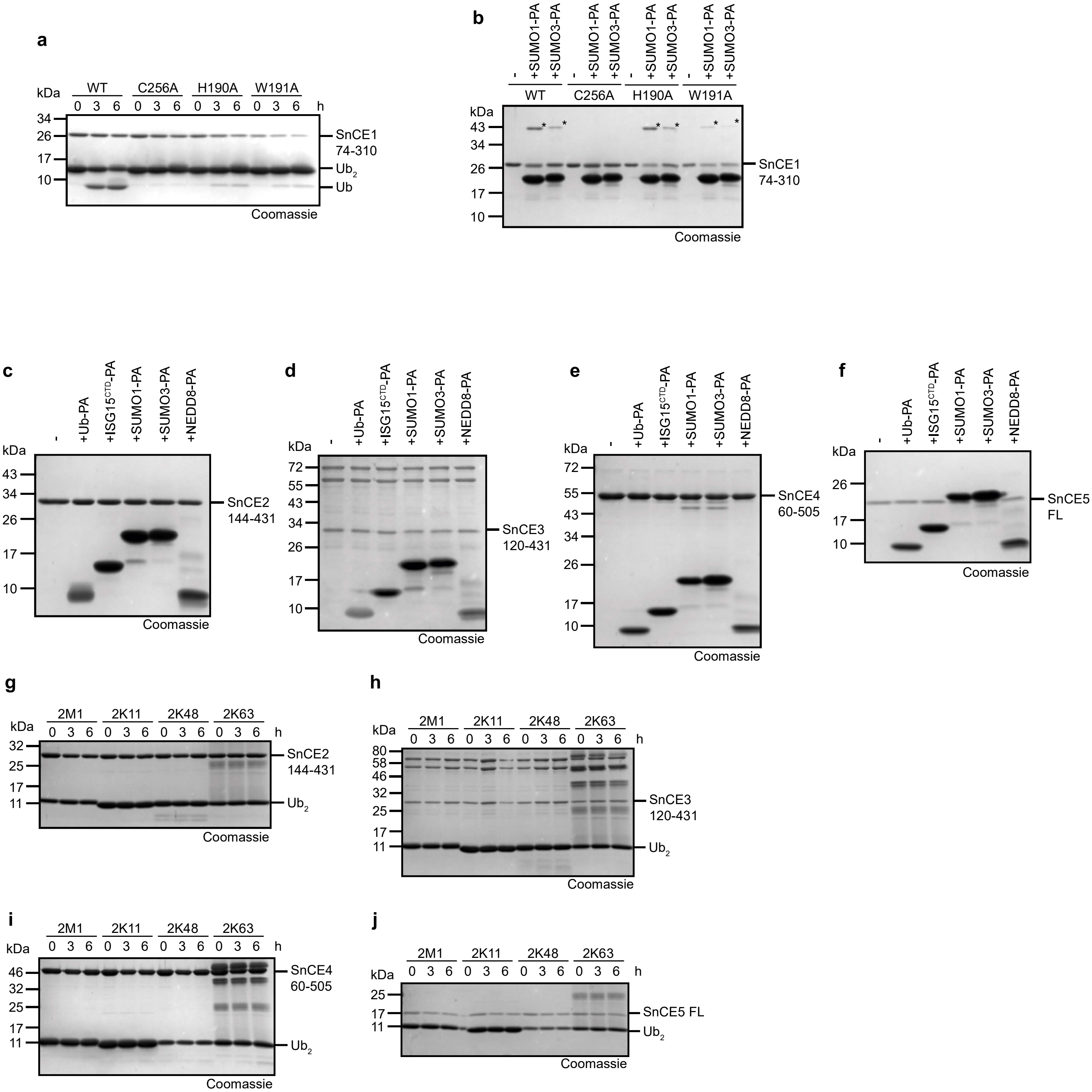


**Supplementary Figure 8**

a, Ubiquitin chain cleavage analysis. K11-linked di-ubiquitin chains were incubated with 5 μM WT enzyme or catalytic/gatekeeper mutants of SnCE1.

b, Activity-based probe reaction of SnCE1 comparing WT enzyme with catalytic and gatekeeper mutants. Asterisks (*) mark the shifted band after reaction.

c-f, Activity-based probe reaction of of SnCE2 (b), SnCE3 (c), SnCE4 (d) or SnCE5 (e) with Ub-PA, ISG15CTD-PA, SUMO1-PA, SUMO3-PA and NEDD8-PA.

g-j, Ubiquitin chain cleavage analysis. A panel of di-ubiquitins was incubated with 5 μM SnCE2 (g), SnCE3 (h), SnCE4 (i) or SnCE5 (j).

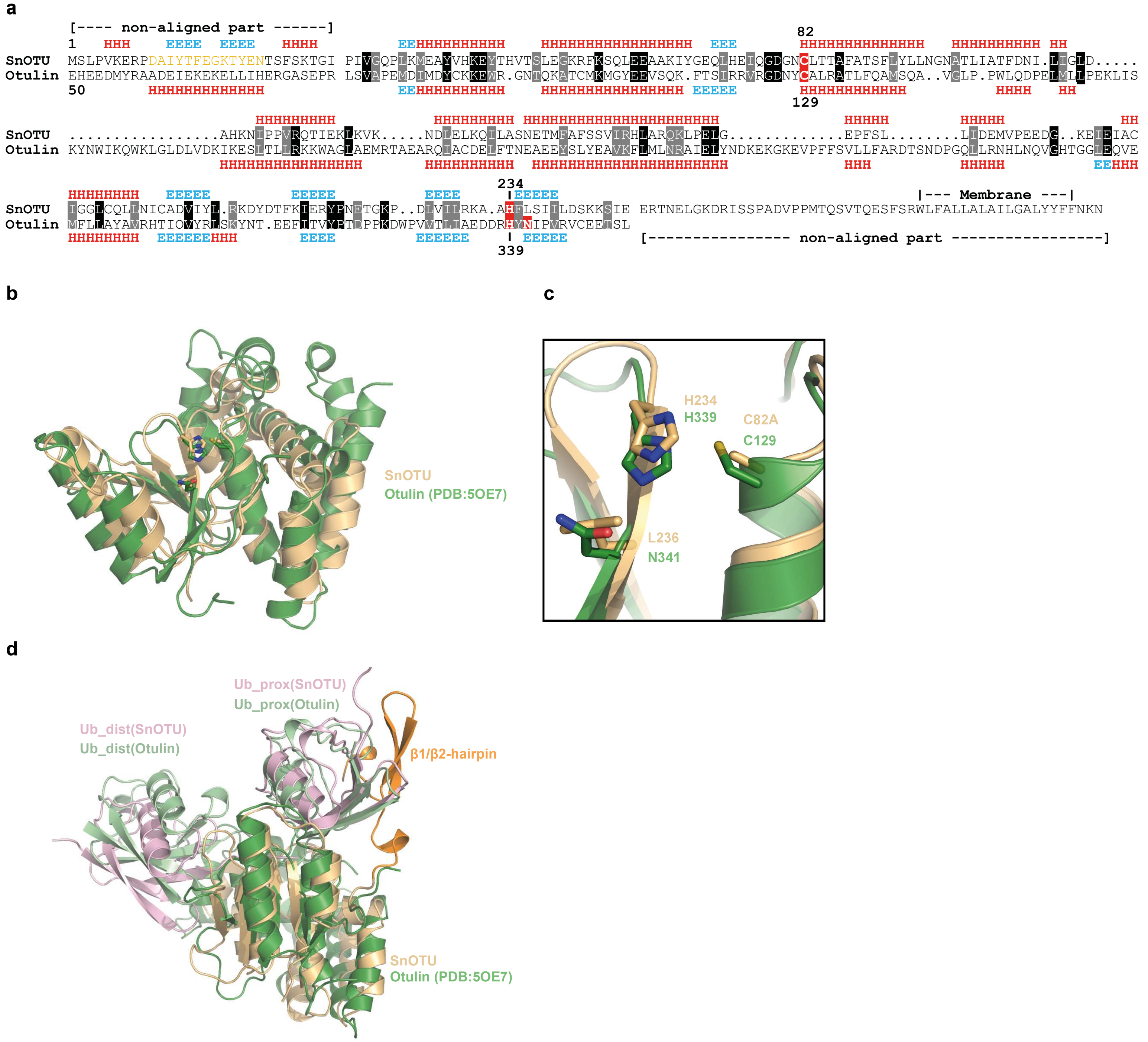


**Supplementary Figure 9**

a,Structurally correct alignment of SnOTU with human Otulin. The central part of the alignment was obtained by structural superposition. Secondary structure elements are shown above and below the alignment. Identical and conserved residues are highlighted on black and grey background, respectively. Catalytically important residues are shown on red background. The β1/β2-hairpin region is colored orange. Secondary structure elements for regions not covered by the available crystal structures were predicted using alphafold models.

b,Structural comparison of SnOTU (orange) and Otulin (PDB: 5OE7, green) are shown in cartoon representation and were superimposed with a RMSD of 3.9 Å over 200 residues.

c, Magnification of the active site residues shown as sticks.

d, Structural comparison of ubiquitin binding by SnOTU and Otulin. The catalytic domains were superimposed as shown in a).

The respective ubiquitins are shown in cartoon representation and colored light pink (SnOTU) and light green (Otulin). The β1/β2-hairpin of SnOTU is highlighted by orange coloring.

**Supplementary Table 1**

**Primers used in this study for cloning and qPCR**

| Amplification/Mutagenesis Primer | | |
|---|---|---|
| | | |
| USP1 | SNE_A12110_167F_pS | GCGAACAGATCGGTGGTTCGGCCCCCGATGATGTTAGTCGCTTTAAGC |
| | SNE_A12110_529R_pS | ATGGTCTAGAAAGCTTTATCTTGGAGTCGATCTAAAAGGTTCTCCTACGCGTTTC |
| | SNE_A12110_C193A_F | CAATAATGAAAACGGGGATGATGCCTTTCTCAATTCCGTGAGACAG |
| | SNE_A12110_C193A_R | CTGTCTCACGGAATTGAGAAAGGCATCATCCCCGTTTTCATTATTG |
| | SnUSP3_H476A_F | AATCAGGAAGCTTATCAGTTGGTGCCTACATTTCCTATGTTTATGACC |
| | SnUSP3_H476A_R | GGTCATAAACATAGGAAATGTAGGCACCAACTGATAAGCTTCCTGATT |
| | SnUSP3_Y477A_F | GGAAGCTTATCAGTTGGTCACGCCATTTCCTATGTTTATGACCG |
| | SnUSP3_Y477A_R | CGGTCATAAACATAGGAAATGGCGTGACCAACTGATAAGCTTCC |
| | | |
| USP2 | SNE_USP2_60F | GCGAACAGATCGGTGGTAAAAATGCCTCTGTTCCCAACTTGAAGATAAGAACAATC ACTATCAATG |
| | SNE_A12380 pOPINS rv | ATGGTCTAGAAAGCTTTAGCCTACTGCTGCTTCTTCAATGCGAGACATACG |
| | USP2_C101A_fw | AGAAACCTCGGAAACACTGCCTGGGGTAACTCAACGC |
| | USP2_C101A_rv | GCGTTGAGTTACCCCAGGCAGTGTTTCCGAGGTTTCT |
| | SnUSP2_H351A_F | GGCACAACCTTAACAAGTGGAGCCTACATTTCACTTGTGAGAAC |
| | SnUSP2_H351A_R | GTTCTCACAAGTGAAATGTAGGCTCCACTTGTTAAGGTTGTGCC |
| | SnUSP2_Y352A_F | CAACCTTAACAAGTGGACACGCCATTTCACTTGTGAGAACCG |
| | SnUSP2_Y352A_R | CGGTTCTCACAAGTGAAATGGCGTGTCCACTTGTTAAGGTTG |
| | | |
| USP3 | SNE_A05310_187F | GCGAACAGATCGGTGGTCATCGCTCAGATTGTCCTCCTCCCTTGAGC |
| | SNE_A05310_522R | ATGGTCTAGAAAGCTTTATGCTTCAAGTTCACTATAAACAAAATAGCAAAAAGATA AGG |
| | USP3 C209A F | GCTGTCTTATAGCATTCATGAAAGCGTCATTCCTGTTCTCATTGACAA |
| | USP3 C209A R | TTGTCAATGAGAACAGGAATGACGCTTTCATGAATGCTATAAGACAGC |
| | SnUSP3_H472A_F | CATGGTGACACAGCTGATAGTGGAGCTTATGTAGCTTATGTTTTTATTCC |
| | SnUSP3_H472A_R | GGAATAAAAACATAAGCTACATAAGCTCCACTATCAGCTGTGTCACCATG |
| | SnUSP3_Y473A_F | GGTGACACAGCTGATAGTGGACATGCTGTAGCTTATGTTTTTATTCC |
| | SnUSP3_Y473A_R | GGAATAAAAACATAAGCTACAGCATGTCCACTATCAGCTGTGTCACC |
| | | |
| Jos1 | SnJos1_1F | GCGAACAGATCGGTGGTATGACTTCCATCTCCTTCAAAGAAAACAATATTGATTCTT TGAATAGTTTCGTTG |
| | SnJos1_398R | ATGGTCTAGAAAGCTTTACAGGAAAGCACAGGGAAGGCTTAACTTTGTGTTTCC |
| | ATX1_C212A_F | GAAGCCCAGAAAAACAGCTTTGCTCAAATGCACGCCGCG |
| | ATX1_C212A_R | CGCGGCGTGCATTTGAGCAAAGCTGTTTTTCTGGGCTTC |
| | SnJos1_H345A_F | CATGCTGGGTACATACCAACCAATTGCTGCTAGTGCGTTA |
| | SnJos1_H345A_R | TAACGCACTAGCAGCAATTGGTTGGTATGTACCCAGCATG |
| | SnJos1_A346W_F | CATGCTGGGTACATACCAACCAATTCATTGGAGTGCGTTACGCAAG |
| | SnJos1_A346W_R | CTTGCGTAACGCACTCCAATGAATTGGTTGGTATGTACCCAGCATG |
| | | |
| Jos2 | SNE_A21910 pOPINS fw | GCGAACAGATCGGTGGTATGAGCGCTATCAATTACACACAAAATAATCTTTTAGGC TTAAACTCTTTTATGC |

| | | |
|---|---|---|
| | SNE_A21910 pOPINS rv | ATGGTCTAGAAAGCTTTACATAAAAGCGCAAGGCAGAGTTAATTTAGAGTTTCCGTTCTG |
| | ATX2_C214A_F | CGAAGAGCAAAAGAACTCCTATGCCCAAATCCATGCTGCAA |
| | ATX2_C214A_R | TTGCAGCATGGATTTGGGCATAGGAGTTCTTTTGCTCTTCG |
| | SnJos2_H347A_F | ATCCTTGGCACATTTATACCTGTAGCTGCCAGAGCTCTCAG |
| | SnJos2_H347A_R | CTGAGAGCTCTGGCAGCTACAGGTATAAATGTGCCAAGGAT |
| | SnJos2_A348W_F | AATCCTTGGCACATTTATACCTGTACATTGGAGAGCTCTCAGAAAAAACAC |
| | SnJos2_A348W_R | GTGTTTTTTCTGAGAGCTCTCCAATGTACAGGTATAAATGTGCCAAGGATT |
| | | |
| | | |
| VTD | SNE_Teg_K74 fw_pK | AAGTTCTGTTTCAGGGCCCGGCACCTGAAAGAGTTTCACTTAAAAAAGGAACTATTTT |
| | SNE_A13000 pOPINS rv | ATGGTCTAGAAAGCTTTATGGTGAGAGCAAGGTCAATGTCATCATTTCAACTTGGTTAG |
| | SNE_C104A_fw | GAAAGGTCAAGGCTTGAACAGTGGCACTTTGTCTTTCTTCTTCTTT |
| | SNE_C104A_rv | AAAAAGAAGAAGAAAGACAAAGTGCCACTGTTCAAGCCTTGACCTT |
| | SnVTD_H275A_F | CAGCATCTGATCTATGATTCTGCTAGTAACACAGCGTTGCATCC |
| | SnVTD_H275A_R | GGATGCAACGCTGTGTTACTAGCAGAATCATAGATCAGATGCTG |
| | | |
| | | |
| OTU | SNE_A17630 pOPINS fw | GCGAACAGATCGGTGGTATGAGCCTTCCTGTCAAAGAACGCCCAGATGC |
| | SNE_A17630 pOPINS rv | ATGGTCTAGAAAGCTTTAGTTTTTGTTGAAAAAGTAGTAGAGTGCACCGAGGATAGCAAG |
| | SnOTU_255R | ATGGTCTAGAAAGCTTTATTTACCAAGCTCATTGGTTCTTTCTTCTATTGATTTTTTTGAGTCAAGAATGATTG |
| | OTU_Y13A_F | CGTAAGTTTTTCCTTCAAATGTAGCGATGGCATCTGGGCGTTCTTTG |
| | OTU_Y13A_R | CAAAGAACGCCCAGATGCCATCGCTACATTTGAAGGAAAAACTTACG |
| | OTU_F15A_F | CGTAAGTTTTTCCTTCAGCTGTATAGATGGCATCTGGGCGTTCTTT |
| | OTU_F15A_R | AAAGAACGCCCAGATGCCATCTATACAGCTGAAGGAAAAACTTACG |
| | OTU_E16A_F | GTATTTTCGTAAGTTTTTCCTGCAAATGTATAGATGGCATCTGGG |
| | OTU_E16A_R | CCCAGATGCCATCTATACATTTGCAGGAAAAACTTACGAAAATAC |
| | OTU_Y20A_F | AAAAAGAGGTATTTTCGGCAGTTTTTCCTTCAAATGTATAGATGGCATCTGG |
| | OTU_Y20A_R | CCAGATGCCATCTATACATTTGAAGGAAAAACTGCCGAAAATACCTCTTTTT |
| | OTU_F25A_F | ACAATAGGGATGCCAGTCTTAGAAGCAGAGGTATTTTCGTAAGTTTTTCC |
| | OTU_F25A_R | GGAAAAACTTACGAAAATACCTCTGCTTCTAAGACTGGCATCCCTATTGT |
| | OTU_I30A_F | TGGCTGGCCAACAATAGGGGCGCCAGTCTTAGAAAAAGAG |
| | OTU_I30A_R | CTCTTTTTCTAAGACTGGCGCCCCTATTGTTGGCCAGCCA |
| | SNE_OTU_H49A_F | TCAAGGCTTGTCACAGCAGTATATTCTTTATGGACATAGGCTTCCA |
| | SNE_OTU_H49A_R | TGGAAGCCTATGTCCATAAAGAATATACTGCTGTGACAAGCCTTGA |
| | OTU_V50A_F | TCCATAAAGAATATACTCATGCGACAAGCCTTGAAGGAAAACG |
| | OTU_V50A_R | CGTTTTCCTTCAAGGCTTGTCGCATGAGTATATTCTTTATGGA |
| | OTU_A50_T51A_F | CATAAAGAATATACTCATGCGGCAAGCCTTGAAGGAAAACGAT |
| | OTU_A50_T51A_R | ATCGTTTTCCTTCAAGGCTTGCCGCATGAGTATATTCTTTATG |
| | OTU_A50_A51_S52A_F | CCATAAAGAATATACTCATGCGGCAGCCCTTGAAGGAAAACGATTTAAG |
| | OTU_A50_A51_S52A_R | CTTAAATCGTTTTCCTTCAAGGGCTGCCGCATGAGTATATTCTTTATGG |
| | OTU_F168A_F | ATTTCGTCAATGAGAAGGCTAGCAGGTTCTCCTAGTTCTGGAAG |
| | OTU_F168A_R | CTTCCAGAACTAGGAGAACCTGCTAGCCTTCTCATTGACGAAAT |

| | | |
|---|---|---|
| | OTU_L170A_F | GGACCATTTCGTCAATGAGAGCGCTAAAAGGTTCTCCTAGTT |
| | OTU_L170A_R | AACTAGGAGAACCTTTTAGCGCTCTCATTGACGAAATGGTCC |
| | OTU_L171A_F | AGGGACCATTTCGTCAATGGCAAGGCTAAAAGGTTCTCCT |
| | OTU_L171A_R | AGGAGAACCTTTTAGCCTTGCCATTGACGAAATGGTCCCT |
| | OTU_E173A_F | CTTCTTCAGGGACCATTGCGTCAATGAGAAGGCTA |
| | OTU_E173A_R | TAGCCTTCTCATTGACGCAATGGTCCCTGAAGAAG |
| | OTU_E183A_F | CGATACAGGCAATCTCAATTGCTTTTCCATCTTCTTCAGGG |
| | OTU_E183A_R | CCCTGAAGAAGATGGAAAAGCAATTGAGATTGCCTGTATCG |
| | OTU_E185A_F | TCCGATACAGGCAATCGCAATTTCTTTTCCATCTTCTTCAGG |
| | OTU_E185A_R | CCTGAAGAAGATGGAAAAGAAATTGCGATTGCCTGTATCGGA |
| | OTU_Y204A_F | CGTAATCTTTACGCAAGGCAATCACATCTGCACAGATGTTTAATAGCTGA |
| | OTU_Y204A_R | TCAGCTATTAAACATCTGTGCAGATGTGATTGCCTTGCGTAAAGATTACG |
| | OTU_R230A_F | AGTGGGCTGCTTTGGCTAAAATGACAAGGTCAGGTTTGCC |
| | OTU_R230A_R | GGCAAACCTGACCTTGTCATTTTAGCCAAAGCAGCCCACT |
| | OTU_K231A_F | TGAGAGAAAGTGGGCTGCTGCGCGTAAAATGACAAGGTCA |
| | OTU_K231A_R | TGACCTTGTCATTTTACGCGCAGCAGCCCACTTTCTCTCA |
| | SNE_OTU_H234A_F | GTCATTTTACGCAAAGCAGCCGCCTTTCTCTCAATCATTCTTGA |
| | SNE_OTU_H234A_R | TCAAGAATGATTGAGAGAAAGGCGGCTGCTTTGCGTAAAATGAC |
| | SnOTU_F235A_F | ATTTTACGCAAAGCAGCCCACGCTCTCTCAATCATTCTTGACTC |
| | SnOTU_F235A_R | GAGTCAAGAATGATTGAGAGAGCGTGGGCTGCTTTGCGTAAAA |
| | | |
| CE1 | CE1_74F_pOPINK | AAGTTCTGTTTCAGGGCCCGTCGCTCACAAGTAAAGCTATTTGTCGCTATTCACTAT CTTCTG |
| | SNE_A10940 pOPINS rv | ATGGTCTAGAAAGCTTTAACAATACAAAGTATTTCCATTTTTTTGTAATTCAAGCAA CTTATCTCGAACTGCAAAG |
| | CE1_C256A_fw | CTTCAACCCGACGGATATCAAGCTGGCCCGTGGG |
| | CE1_C256A_rv | CCCACGGGCCAGCTTGATATCCGTCGGGTTGAAG |
| | SnCE1_H190A_F | CCTATCCTCTTTTCATCGGGGGAAATGCTTGGGGACTACTAT |
| | SnCE1_H190A_R | ATAGTAGTCCCCAAGCATTTCCCCCGATGAAAAGAGGATAGG |
| | SnCE1_W190A_F | CTCTTTTCATCGGGGGAAATCATGCGGGACTACTATTTATCG |
| | SnCE1_W190A_R | CGATAAATAGTAGTCCCGCATGATTTCCCCCGATGAAAAGAG |
| | | |
| | | |
| CE2 | SNE_5_CE_430_fw | GCGAACAGATCGGTGGTACCAATAGGGCATTTTTCTTACCTGATGCTGATGATCTA ACTG |
| | SNE_A13010 pOPINS rv | ATGGTCTAGAAAGCTTTACCATGATCGTGGATCATGAGATAAGTCAACGTCATAAG G |
| | | |
| CE3 | SNE_7CE_popinS_120_F | GCGAACAGATCGGTGGTTCTTTTCCGGCTACTCCTCAAACTTCCACCACTTC |
| | SNE_A19290 pOPINS rv | ATGGTCTAGAAAGCTTTAGGGCGTGCCTTCGGTAAATTTTTTAAATTGGCTCTCATG |
| | | |
| CE4 | SNE_6_CE_178_fw | GCGAACAGATCGGTGGTCACATTGCGGAGAATTGGAATGCAATTTGGTCATCACC |
| | SNE_A14650 pOPINS rv | ATGGTCTAGAAAGCTTTAAGATAAATCTGCAATGAGTTCCAATCGAATCTCTTTTGG CTTCTTC |
| | | |
| CE5 | SNE_A22800 pOPINS fw | GCGAACAGATCGGTGGTATGGATCTTTTAGAGAAGCAGTACGGTGCAAAAGCTAC ATTTATTC |
| | | |

| | | |
|---|---|---|
| qPCR Primer | | |
| | SNE_A12110_F | GCTTAACAGTGGGCGTGTTG |
| | SNE_A12110_R | AGAGAAGGTGCTCAGGAGGT |
| | SNE_A12380_F | CGGAAACACTTGCTGGGGTA |
| | SNE_A12380_R | TGGCTCAGATTTCCGTTGCT |
| | SNE_A05310_F | ACTTGAGTTGGGTGTTCGCT |
| | SNE_A05310_R | TACGCGGATTTGACAGAGCA |
| | SNE_A21920_F | CCTTTCTGGGATATGGCGCT |
| | SNE_A21920_R | GCCATCAGCAGGGTCTTCTA |
| | SNE_A21910_F | GGGACCTTAGACGAACGGAC |
| | SNE_A21910_R | ACTGTTCCTCCACCCGAGTA |
| | SNE_A13000_F | TTTACCCTCTTGAGCGAGCG |
| | SNE_A13000_R | TGGGTGAACCAGGATTTGCT |
| | SNE_A17630_F | CTGATGTGCCCCCAATGACT |
| | SNE_A17630_R | GAGCGAGCAGTGCAAATAGC |
| | SNE_A10940_F | AACACGACGGTATCCAAGCA |
| | SNE_A10940_R | AGAGCGTCCAAATCAACCCA |
| | SNE_A13010_F | TCGGCAAGTAGGAACAACGA |
| | SNE_A13010_R | GGTTTTGATTCGATGGGCGG |
| | SNE_A19290_F | GGAAGTGCCAATGGATGCTC |
| | SNE_A19290_R | TGCGCCGTCAAAAATGATGT |
| | SNE_A14650_F | ACTGCAAAGGTCGCTTCGTA |
| | SNE_A14650_R | AAGCCCTTCGATTCCTGAGC |
| | SNE_A22800_F | TGCTTCTTCAACTACAGGCGT |
| | SNE_A22800_R | AGAGAAGCAGTACGGTGCAA |
| GroEL C | SNE_A22590_F | TTCCATCACCGGCAACATCA |
| | SNE_A22590_R | GCAAAAGAGATCGCGCTGAA |
| 5S RNA Simkania | SNE_Ar0030_F | CACCTGATCCCATCCCGAAC |
| | SNE_Ar0030_R | GCGACCTACTCTCCCGTACT |
| | GAPDH_F | GTCTCCTCTGACTTCAACAGCG |
| | GAPDH_R | ACCACCCTGTTGCTGTAGCCAA |
| 5S RNA Amoebal | 5S RNA_F | ATACTGCGCAGAAAGCAC |
| | 5S RNA_R | ACCCCAGTACTAACACCG |
| | 18SQV_F | CCCAGATCGTTTACCGTGAA |
| | 18SQV_R | TAAATATTAATGCCCCCAACTATCC |
| | HPRT_F | GGAGCGGATCGTTCTCTG |
| | HPRT_R | ATCTTGGCGTCGACGTGC |
