# Supplementary material for: Functional and structural diversity in deubiquitinases of the Chlamydia-like bacterium Simkania negevensis
Source: Nat Commun. 2023 Nov 13;14:7335. doi: 10.1038/s41467-023-43144-y (PMC10643670; doi:10.1038/s41467-023-43144-y)

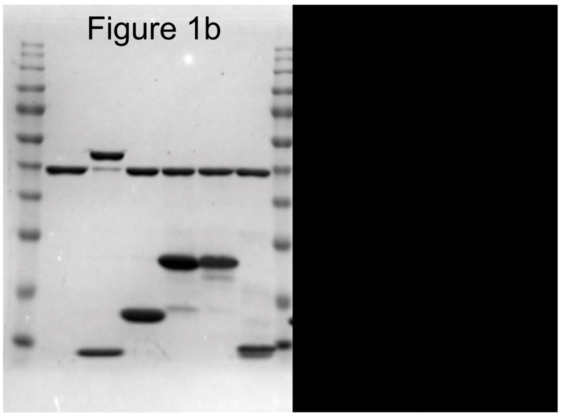
Figure 1b

not used in this study

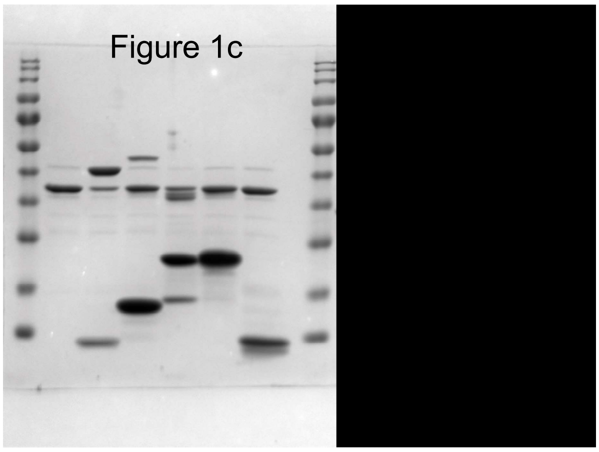
Figure 1c

not used in this study

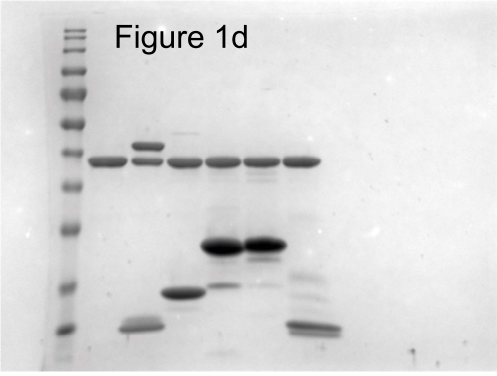
Figure 1d

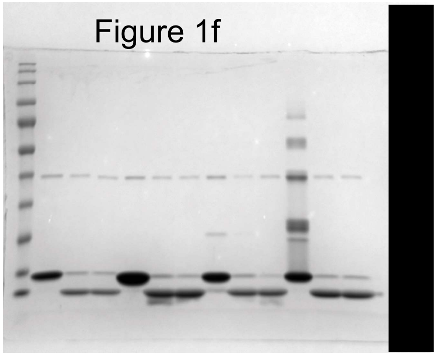
Figure 1f

not used in this study

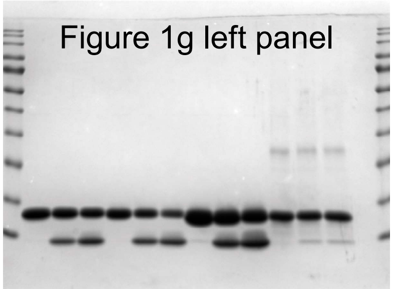
Figure 1g left panel

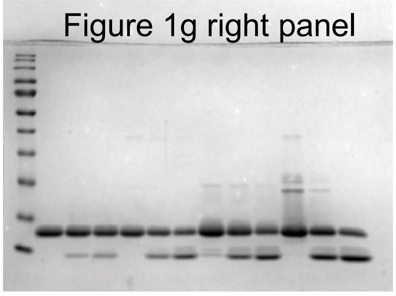
Figure 1g right panel

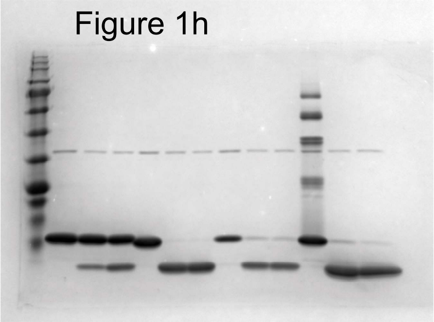
Figure 1h

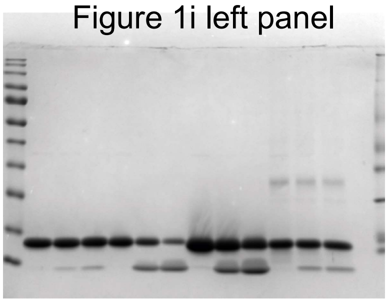
Figure 1i left panel

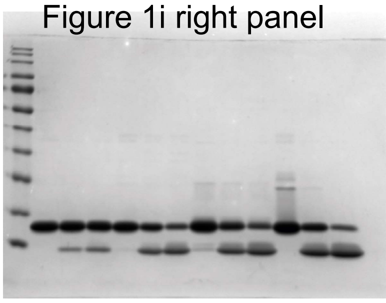
Figure 1i right panel

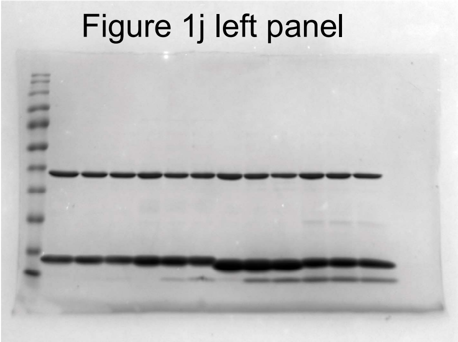
Figure 1j left panel

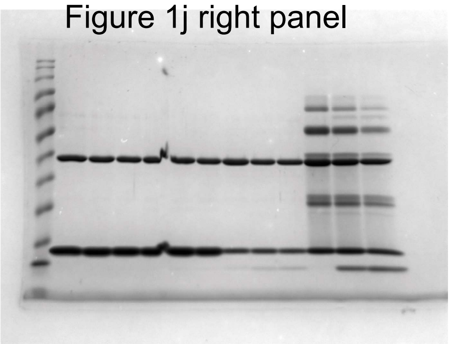
Figure 1j right panel

Figure 2b

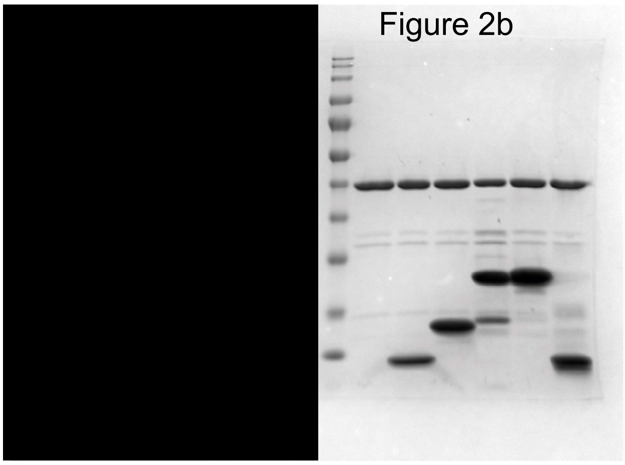

not used in this study

Figure 2c

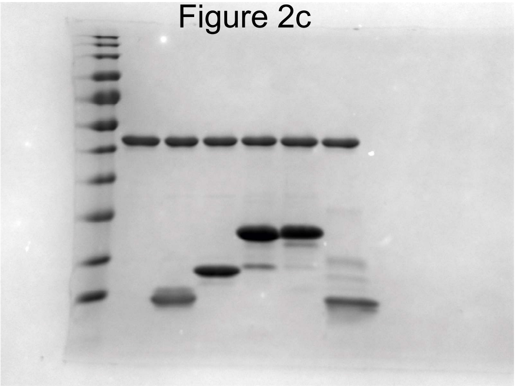

Figure 2e left panel

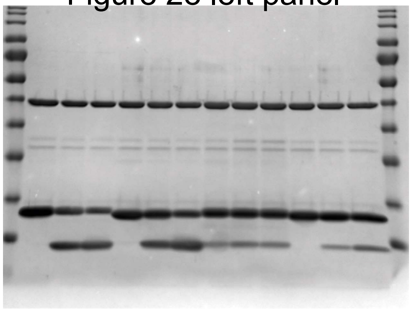

Figure 2e right panel

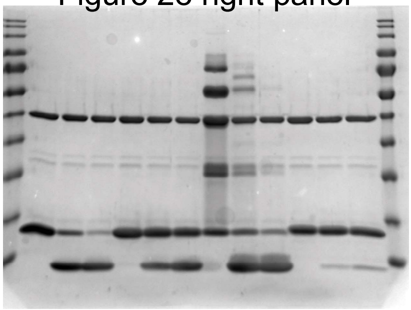

Figure 2f left panel

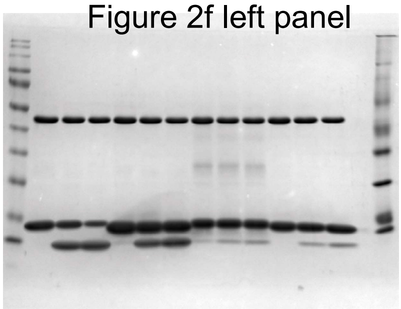

Figure 2f right panel

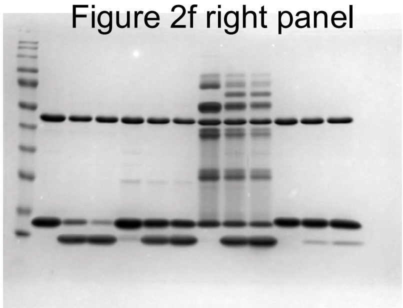

Figure 3b

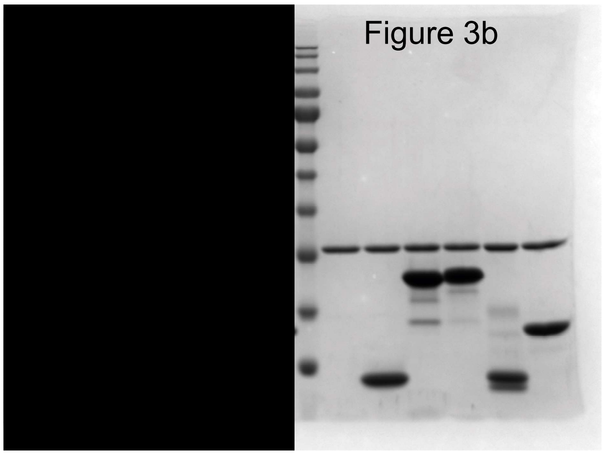

not used in this study

Figure 3d left panel

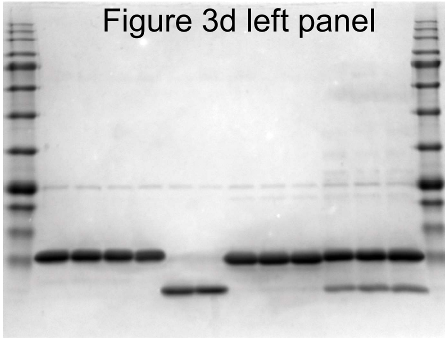

Figure 3d right panel

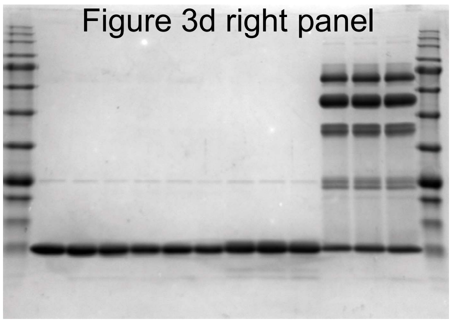

Figure 4b

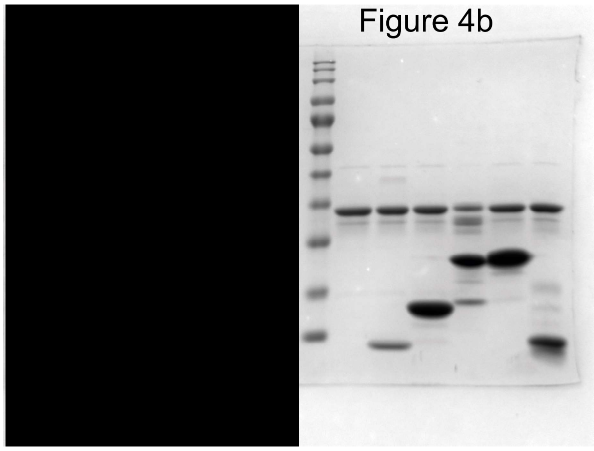

[redacted]

not used in this study

Figure 4d left panel

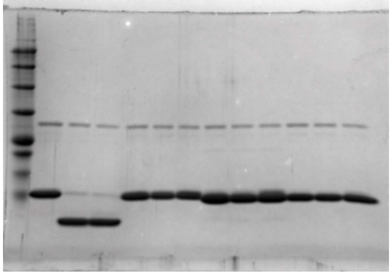

Figure 4d right panel

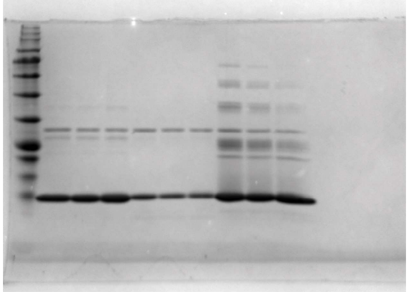

Figure 5f left panel

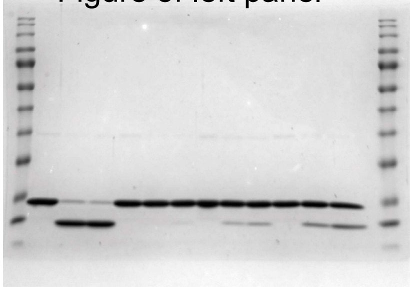

Figure 5f right panel

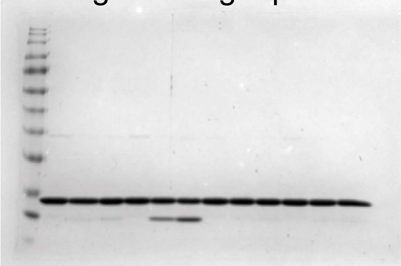

Figure 5g

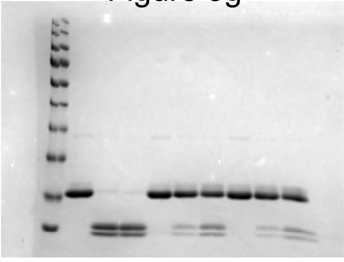

Figure 5j left panel

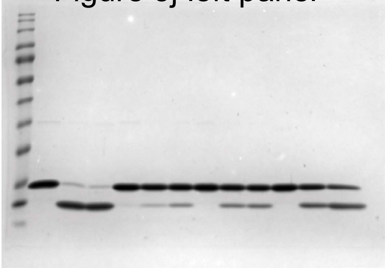

Figure 5j right panel

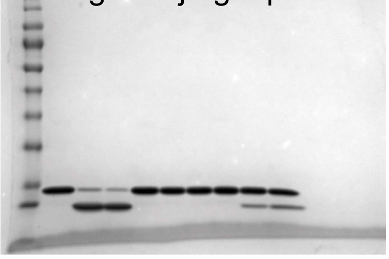

Figure 5k

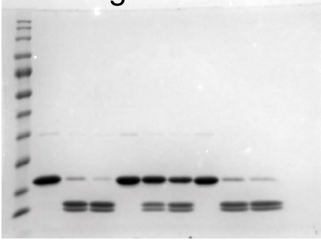

Figure 6b

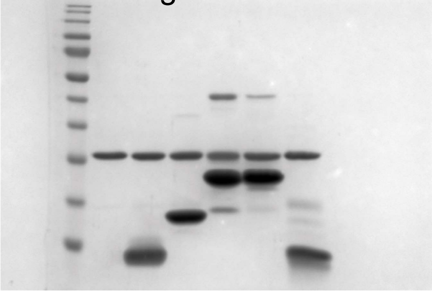

d

Figure 6d left panel

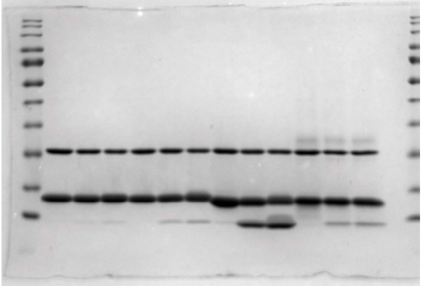

Figure 6d right panel

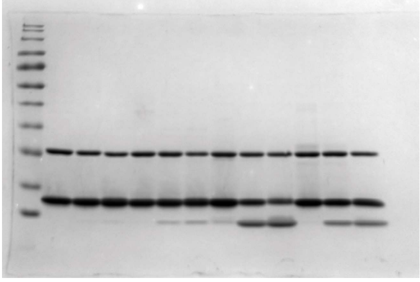

Figure 6e

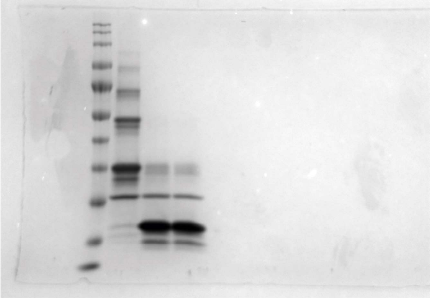

Supplement: Supplementary file 4 — Source Data [file 41467_2023_43144_MOESM4_ESM.zip › Raw_Data/raw_images_mainfigures.pdf]
