# Supplementary material for: Functional and structural diversity in deubiquitinases of the Chlamydia-like bacterium Simkania negevensis
Source: Nat Commun. 2023 Nov 13;14:7335. doi: 10.1038/s41467-023-43144-y (PMC10643670; doi:10.1038/s41467-023-43144-y)

Supplementary Figure 3a

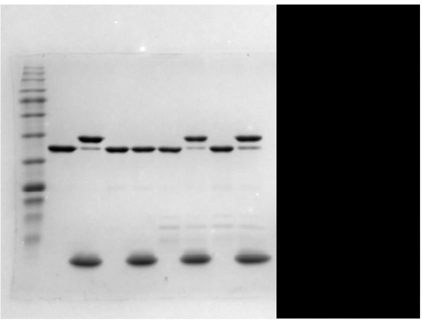

not used in this study

Supplementary Figure 3b

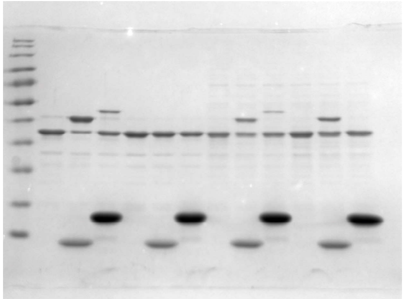

Supplementary Figure 3c

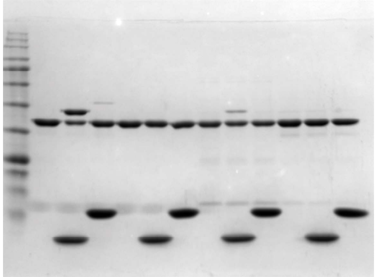

Supplementary Figure 3d

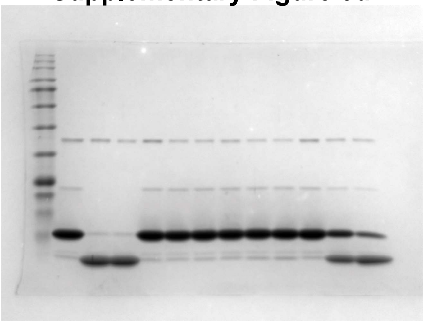

Supplementary Figure 3e

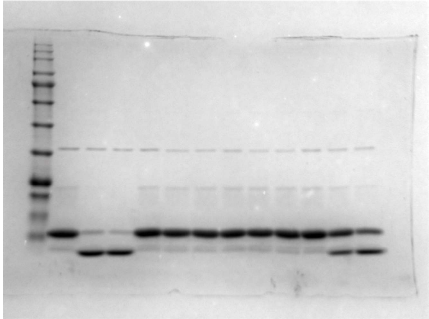

Supplementary Figure 3f

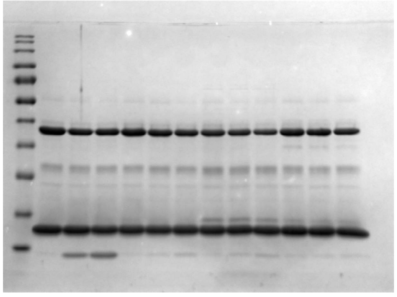

Supplementary Figure 4a

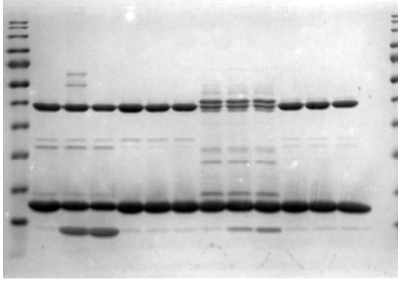

Supplementary Figure 4b

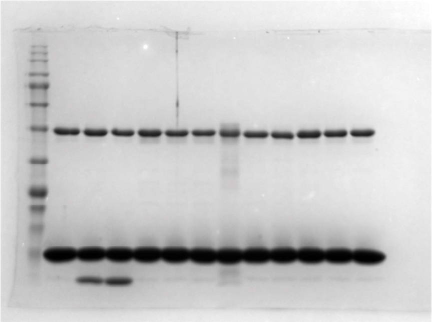

Supplementary Figure 4c

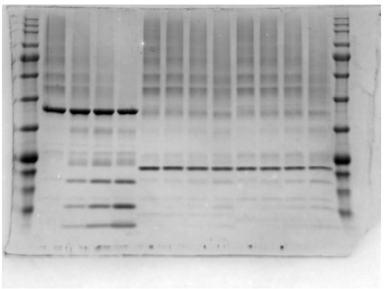

Supplementary Figure 5a

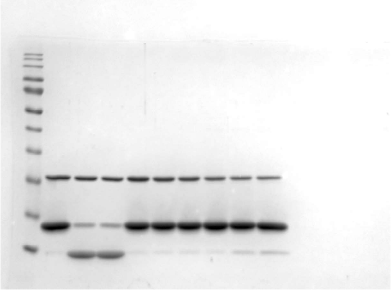

Supplementary Figure 6a

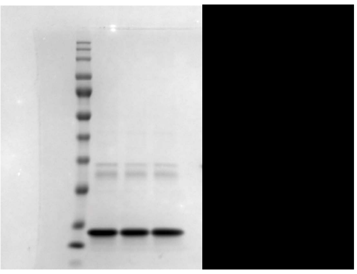

not used in this study

b

Supplementary Figure 6b

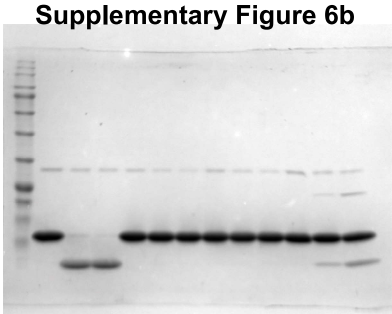

Supplementary Figure 6c left panel

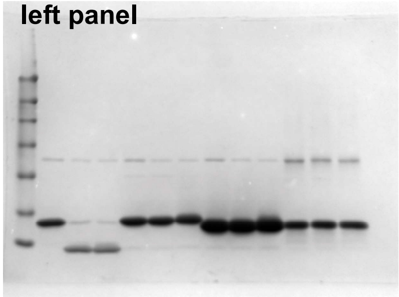

Supplementary Figure 6c right panel

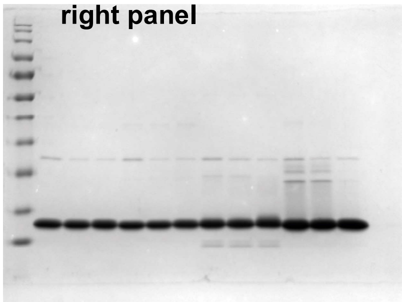

Supplementary Figure 6d

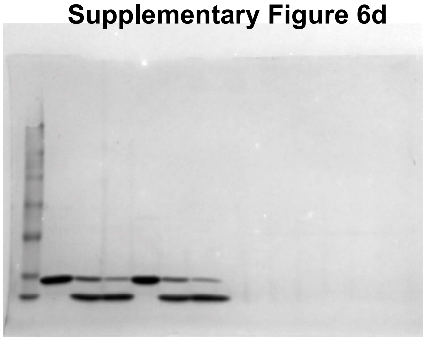

Supplementary Figure 7b

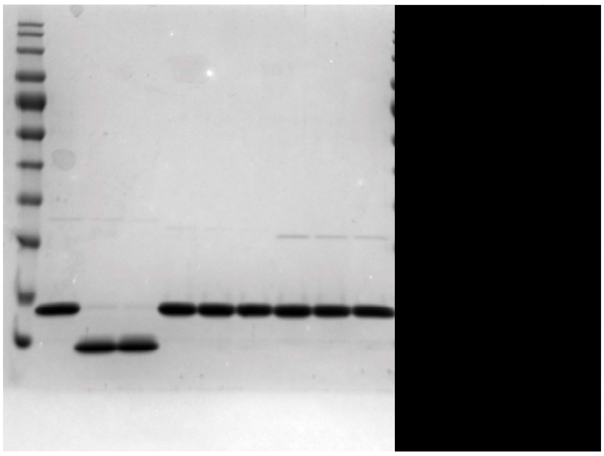

not used in this study

Supplementary Figure 7d

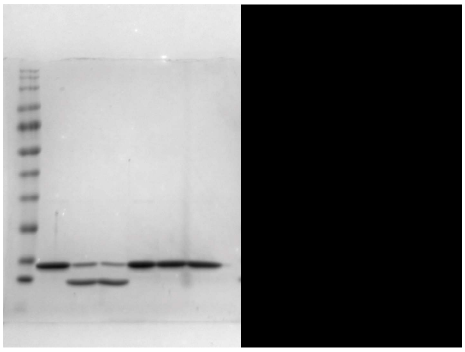

not used in this study

Supplementary Figure 7e

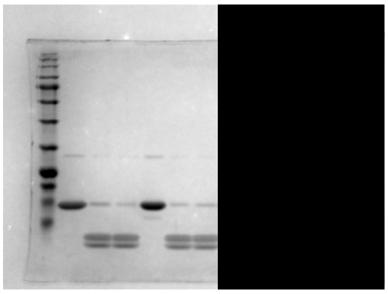

not used in this study

Supplementary Figure 7f

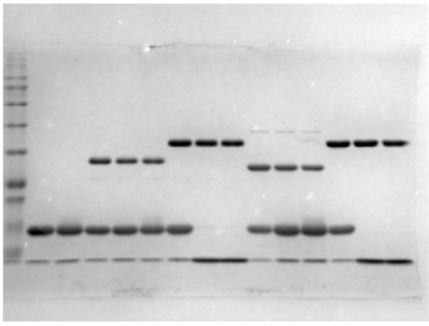

Supplementary Figure 7g

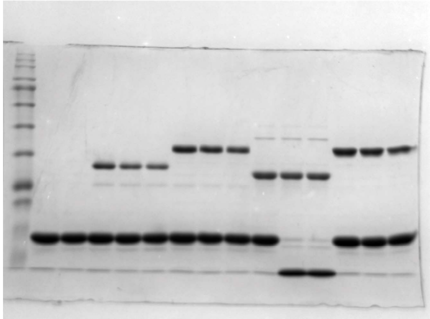

Supplementary Figure 8a

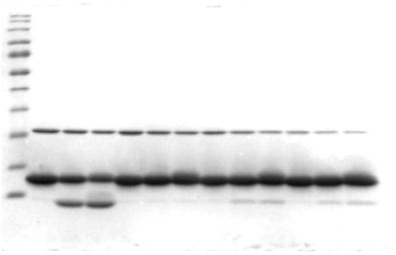

Supplementary Figure 8b

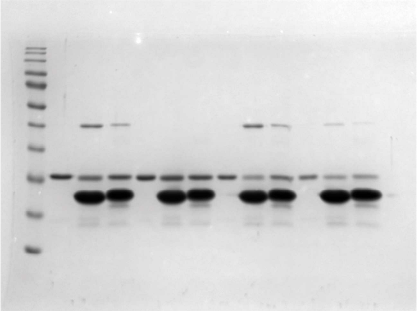

Supplementary Figure 8c

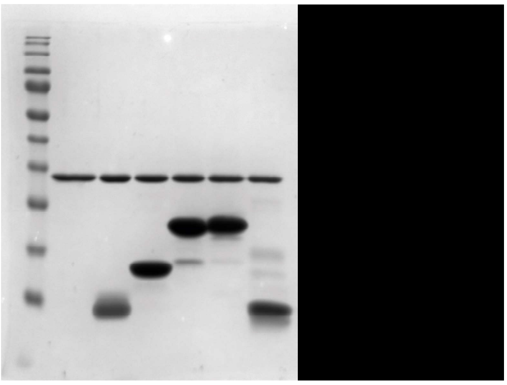

not used in this study

Supplementary Figure 8d

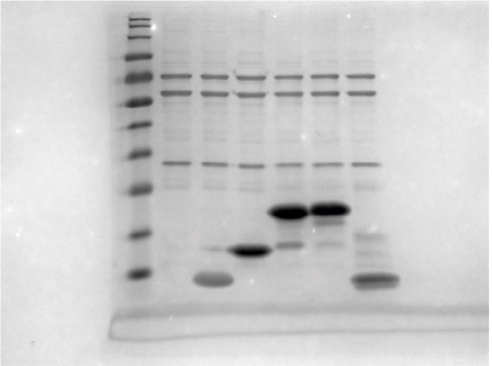

Supplementary Figure 8e

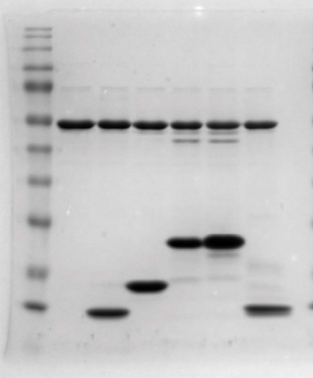

Supplementary Figure 8f

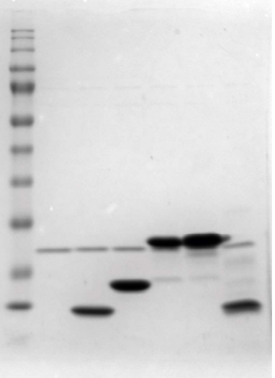

Supplementary Figure 8g

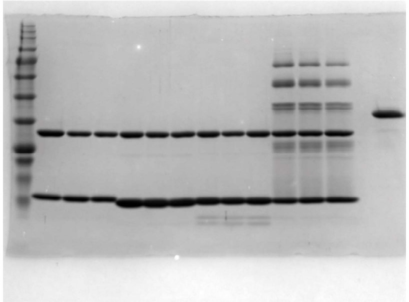

Supplementary Figure 8h

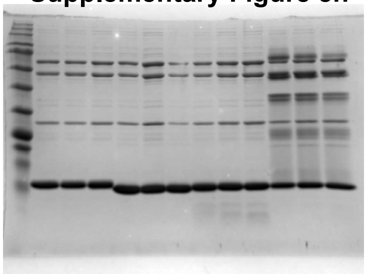

Supplementary Figure 8i

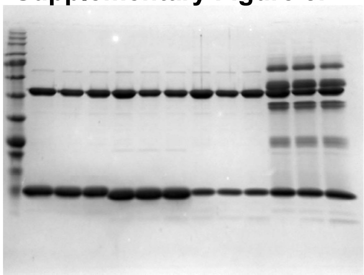

Supplementary Figure 8j

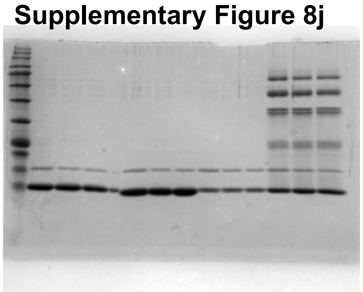

Supplement: Supplementary file 4 — Source Data [file 41467_2023_43144_MOESM4_ESM.zip › Raw_Data/Raw_images_suppfigures.pdf]
